# Predictive value of small dense low-density lipoprotein cholesterol for cardiovascular events in Chinese elder diabetes mellitus patients

**DOI:** 10.1186/s13098-021-00667-y

**Published:** 2021-04-28

**Authors:** Li Xu, Xu Chen, Jingfen Lu, Yan Xu, Honglin Yang, Xuewen Zhou, Jun Zhou, Jianhong Xu, Hao Shen

**Affiliations:** 1 Department of General Surgery, Suzhou Ninth People’s Hospital, Soochow University, Suzhou, China; 2 Department of Nephrology, Suzhou Ninth People’s Hospital, Soochow University, Suzhou, China; 3 Department of Blood Transfusion and Clinical Laboratory Medicine, Suzhou Ninth People’s Hospital, Soochow University, Suzhou, China; 4 Department of Emergency Medicine, Suzhou Ninth People’s Hospital, Soochow University, Suzhou, China

**Keywords:** Cardiovascular event, Small dense low-density lipoprotein cholesterol, Receiver operating characteristic curve, Diabetes mellitus

## Abstract

**Background:**

As a subcomponent of low-density lipoprotein cholesterol (LDL-C), small dense LDL-C (sdLDL-C) has been suggested to be a better predictor of cardiovascular diseases (CVD). The aim of this research was to evaluate the predictive value of the sdLDL-C in cardiovascular events (CVs) in Chinese elderly patients with type 2 diabetes mellitus (DM).

**Methods:**

A total of 386 consecutive type 2 DM patients were included into this study during December 2014 to December 2016. The serum sdLDL-C level of each subject was measured by homogeneous method. During a period of 48-month’s follow-up, the occurrence of CVs and associated clinical information were recorded. Receiver operating characteristic (ROC) curves were used to assess the predictive value of serum sdLDL-C to occurrence of major CVs.

**Results:**

A total of 92 CVs occurred during the study period. The ROC curve analysis manifested that sdLDL-C in the study population had a matchable discriminatory power (AUC for sdLDL-C was 0.7366, P = 0.003). In addition, Kaplan-Meier event-free survival curves displayed an obvious increase of CVs risk for sdLDL‐C ≧ 26 mg/dL (log-rank = 9.10, P = 0.003). This phenomenon had analogous results in patients who received statins at baseline (log rank = 7.336, P = 0.007). Cox regression analysis revealed that the increase in HbA1c, glucose, LDL-C, sdLDL-C, non-high-density lipoprotein cholesterol (non-HDL-C) and apolipoprotein B (ApoB) and the decrease in apolipoprotein AI (ApoAI) were obviously interrelated with heightened CVs risk. Multiple Cox regression demonstrated that the increase of sdLDL-C and hemoglobin A1c (HbA1c) was significantly correlated with CVs. The results of the study indicated that high sdLDL-C level (> 10 mg/dL) was a risk factor for CVs in the multivariate model (HR 1.281, 95% CI 1.225–16.032; P < 0.01).

**Conclusion:**

sdLDL-C level could be an effective predictor in predicting the future CVs for Chinese elderly patients with type 2 DM and dyslipidemia.

## Background

Type 2 diabetes mellitus (DM) patients are more likely to have cardiovascular disease (CVD) partially due to dyslipidemia characterized by elevated low-density lipoprotein cholesterol (LDL-C), small dense LDL-C(sdLDL-C), remnant lipoprotein cholesterol (RLP-C) and decreased HDL-C in serum [1, 2]. Moreover, previous researches had discovered that higher sdLDL-C and RLP-C levels were associated with elevated risk of CVD events [3, 4]. Moreover, serum levels of lipoprotein(a) (Lp(a)) can be risk factors for adverse events [5].

A cross-sectional study has shown that sdLDL-C concentration was closely related to the severity of cardiovascular disease and was independent of classic coronary risk factors [6]. Moreover, another study has revealed that a higher RLP-C concentration was a risk factor for cardiovascular events (CVs) independent of other risk factors in diabetic patients [7]. However, few studies in the past have assessed the relationship between sdLDL-C and RLP-C and CVs in Chinese elderly patients with type 2 diabetes mellitus (DM).

The serum sdLDL-C level could be easily detected through automated analysis. However, these methods have not yet been impressed on large number of type 2 DM patients, especially among the Chinese elderly population. The study attempted to investigate whether sdLDL-C can predict CVs or sdLDL-C as a complement to the routine lipid profile should be considered in clinical practice in order to improve the management of CVD and risks factors for their progression in Chinese elderly patients with type 2 DM [8].

## Methods

### Subjects and study design

This research included 418 consecutive patients aged ≥ 65 years with type 2 DM at Suzhou Ninth People′s Hospital, Suzhou, China, between December 2014 and December 2016, and all of the subjects had no history of CVD. DM was identified according to 2009 American Diabetes Association Criteria for diabetes diagnosis [9]. The body mass index (BMI), estimate of glomerular filtration rate (eGFR) and smoking status (current smokers and at least one cigarette per day) were recorded. The diagnosis of hypertension was based on blood pressure measurement (systolic blood pressure ≥ 140 mmHg and/or diastolic blood pressure ≥ 90 mmHg) and/or use of antihypertensive medications within 2 weeks of enrollment [10]. Dyslipidemia was defined as the fasting serum LDL-C ≥ 140 (mg/dL), high-density lipoprotein cholesterol (HDL-C) < 40 (mg/dL) or triglyceride (TG) ≥ 150 (mg/dL) and/or the current use of lipid-lowering medication [11]. Exclusion criteria were: age ≥ 90 years (*n* = 2), presence of malignancy (*n* = 3), known thyroid disorders (*n* = 3), lost of blood examination data (*n* = 6), infectious disease (*n* = 7), lost during follow-up (*n* = 7) and severe hepatic and nephrotic diseases (*n* = 4).

Finally, a total of 386 patients (mean age of 72.7 ± 5.4 year, range from 65 to 86 years) were included into the study (269 males and 117 females). They received follow-ups ranging from 20 to 48 months, with an average of 28 months. Blood biochemical examinations were performed for subjects yearly during the follow-up period. The associated clinical and laboratory data was collected between September and November 2019. The endpoints were: (1) CVD death, (2) the date of the first occurrence of CVs, and (3) the date of the patient′s last visit to Suzhou Ninth People′s Hospital. CVs were defined as (1) death caused by cardiovascular disease, (2) onset of acute coronary syndrome, (3) congestive heart failure, (4) need for coronary or other arterial revascularization, (5) stroke and registered in Table 1.

**Table 1:** Cardiovascular events were defined and registered during the follow-up

Cardiovascular events defined	Registered (n = 92)
Death caused by cardiovascular disease	8
Onset of acute coronary syndrome	13
Congestive heart failure	26
Need for coronary or other arterial revascularization	36
Stroke	9

### Laboratory measurements

The blood samples were collected after 12 h of fasting in the morning. After collection, the samples were centrifuged immediately and stored at – 80 ℃ until assay within the same day. High-sensitivity C-reactive protein (hsCRP), HDL-C, Hemoglobin A1c (HbA1c), LDL-C, TG, fasting blood glucose, apolipoprotein A-I(ApoA-I), apolipoprotein B(ApoB) and lipoprotein (a) were detected through standard biochemical tests as reported previously [12]. The sdLDL-C and RLP-C were detected by a detergent-based fully-automatic homogeneous method (Denka Seiken kit, Tokyo, Japan) [13].

### Statistical analyses

All statistical analyses were performed using the SAS 9.1 software package. The Chi-square test was used to analyze categorical variables. Wilcoxon test and independent student *t*-test was used to compare the means between the CVs group and non-CVs group. Correlation coefficients between sdLDL-C and other parameters were determined by Spearman's rank analysis. Kaplan-Meier method was used to compare the occurrence of CVs between the lower sdLDL-C and higher sdLDL-C group, and the differences were assessed with log-rank test. The receiver operating characteristic (ROC) curves and area under the curve (AUC) were used for assess the ability of sdLDL-C, HbA1c, and RLP-C to predict CVs. Cox regression and multivariate Cox regression analysis were employed to determine these independent predictors. All statistical analyses were double-tailed, and statistical significance was considered at the level of P < 0.05.

## Results

The occurrences of CVs were summarized in Table 1. The patients with CVs had obviously higher BMI and were more likely to be under calcium channel blockers and insulin therapy compared with non-CVs patients (Table 2). A comparison of laboratory findings manifested the levels of glucose, HbA1c, LDL-C, non-HDL-C, sdLDL-C, RLP-C, ApoB and ApoA-1 in the CVs group were significantly different from those of the non-CVs group (Table 3). Whereas there was no statistically significant difference in TG, hsCRP, HDL-C, eGFR, sdLDL-C/LDL-C ratioand lipoprotein (a) between the two groups.

**Table 2:** The clinical characteristics of the enrolled patients at baseline

Variables	Whole	CVs	Non-CVs	*P*^a^
	(*n* = 386)	(*n* = 92)	(*n* = 294)	
Characteristics				
Age (years)	72.7 ± 5.4	72.9 ±5.2	72.8 ± 5.5	0.568
Male	269/386 (69.7)	65/92 (70.6)	204/294 (69.4)	0.818
BMI (kg/m^2^)	23.6 ± 2.1	24.3 ± 2.3	23.4 ± 2.2	0.001
Cardiovascular disease risk factors				
Hypertension	270/386 (69.9)	64/92 (69.6)	206/294 (70.1)	0.927
Dyslipidemia	246/386 (90.2)	83/92 (90.2)	265/294 (90.1)	0.982
Smoking, current or former	246/386 (63.7)	59/92 (64.1)	187/294 (63.6)	0.927
Family history	82/386 (21.2)	21/92 (22.8)	61/294 (20.7)	0.671
Medications				
Calcium-channel blocker	165/386 (42.7)	48/92 (52.2)	117/294 (39.8)	0.036
ACEI	212/386 (54.9)	56/92 (60.9)	156/294 (53.1)	0.189
ARB	172/386 (14.8)	40/92 (43.5)	132/294 (44.9)	0.943
* β*-blocker	114/386 (44.5)	27/92 (29.3)	87/294 (29.6)	0.964
Aspirin	347/386 (89.9)	84/92 (91.3)	263/294 (89.5)	0.608
Insulin	27/386 (7.0)	12/92 (13.0)	15/294 (5.1)	0.009
Sulfonylurea	147/386 (40.0)	74/92 (80.0)	73/294 (25.0)	0.001
Metformin	38/386 (10.0)	9/92 (9.7)	29/294 (10.0)	0.999
α-Glucosidase inhibitor	127/386 (33.0)	54/92 (58.6)	73/294 (25.0)	0.001
Thiazolidine	69/386 (18.0)	49/92 (53.2)	20/294 (6.8)	0.001
Statin	224/386 (58.0)	47/92 (51.1)	162/294 (55.1)	0.501

Data are presented as mean ± SD or the number and its percentage (%) percentage = the number of each individual category divided by n CVs, cardiovascular events; BMI: body mass index; ACEI: angiotensin-converting enzyme inhibitor; ARB, angiotensin-receptor blocker

^a^Indicates the comparison of mean or percentage between CVs group and non-CVs group

**Table 3:** The laboratory characteristics of the enrolled patients at baseline

Variables	Whole	CVs	Non-CVs	*P*^a^
	(n =386)	(n =92)	(n = 294)	
Triglycerides (mg/dL)	123.6 ± 71.3	134.5 ± 88.3	120.3 ± 63.2	0.097
LDL-C (mg/dL)	107.9 ± 30.5	116.7 ± 29.9	105.1 ± 29.8	0.001
sdLDL-C (mg/dL)	31.0± 12.1	36.2± 15.2	29.4± 16.8	0.001
HDL-C (mg/dL)	46.8± 15.1	44.2 ± 12.9	47.6 ± 15.1	0.052
Non-HDL-C (mg/dL)	129.3± 34.1	138.3±35.5	126.5± 33.4	0.004
sdLDL-C/LDL-C	0.29 ± 0.11	0.31 ± 0.13	0.28 ± 0.16	0.102
RLP-C (mg/dL)	5.0± 3.1	5.6± 3.5	4.8± 2.7	0.022
Glucose (mg/dL)	114.9 ±34.5	122.6 ±46.4	112.5 ± 29.5	0.014
HbA1c (%)	6.38 ± 1.2	6.8 ± 1.3	6.25 ±1.09	0.001
ApoA-I (mg/dL)	125.0 ± 25.3	119.3 ± 23.6	126.8 ± 27.1	0.018
ApoB (mg/dL)	86.1 ± 21.3	92.1 ± 21.6	84.2 ± 22.1	0.003
eGFR (mL/min/1.73 m^2^)	69.9 ± 19.2	68.8 ± 14.1	70.3± 15.6	0.411
hsCRP (mg/dL)	0.54 ± 1.1	0.53 ± 1.3	0.55 ±0.98	0.875
Lp (a) (mg/dL)	22.9 ± 24.1	25.1 ± 25.2	22.3 ±24.1	0.401

Data are presented as mean mean ± SD. LDL-C: low-density lipoprotein cholesterol; sdLDL-C: small dense low-density lipoprotein cholesterol; HDL-C: high-density lipoprotein cholesterol; RLP-C: remnant lipoprotein cholesterol; HbA1c: hemoglobin A1c; ApoA-I: apolipoprotein A-I; ApoB: apolipoprotein B; eGFR: estimated glomerular filtration rate; hsCRP: high-sensitivity C-reactive protein; Lp (a): lipoprotein (a)

^a^Indicates the comparison of mean or percentage between CVs group and non-CVs group

**Fig. 1:**
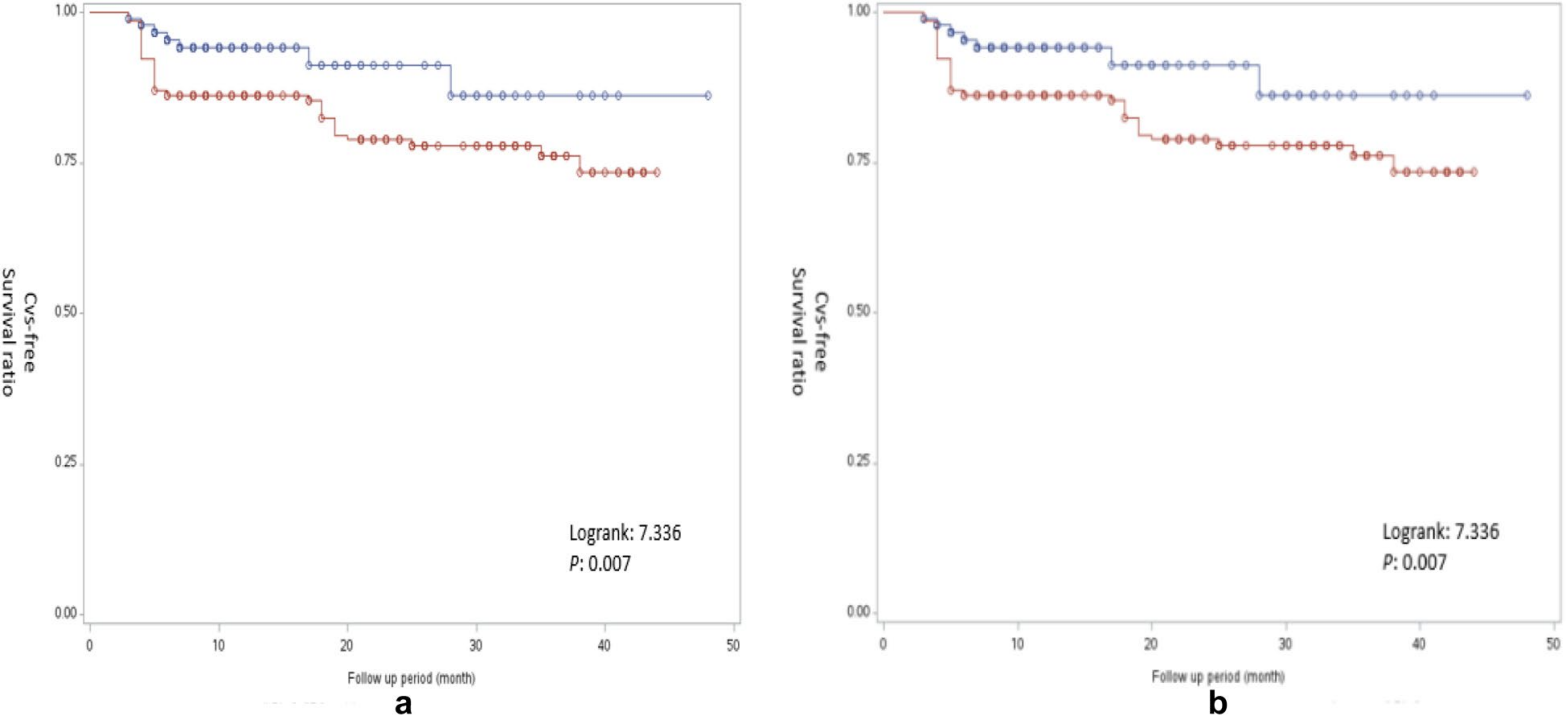
The Kaplan–Meier event free survival of patients stratified by the median small dense low-density lipoprotein cholesterol (sdLDL-C) concentration (26 mg/dL). **a** All patients, **b** patients treated with statins

In this study, first-time CVs were observed in 92 patients. Kaplan-Meier method showed an obvious increase of CVs risk for the median levels of sdLDL‐C (Fig. 1a). Similar trend was found in patients who received statins (Fig. 1b). Cox regression analysis showed that increase in sdLDL-C and HbA1c was associated with a higher risk for CVs (Table 4). To determine whether sdLDL-C was an independent risk factor, we performed Cox multivariate regression analysis. The models were built after adjustment age, gender and CVs risk factors. Model 1 (including Glucose, HbA1c, LDL-C, Non-HDL-C, sdLDL-C, ApoA-I and ApoB) and Model 2 (only including sdLDL-C and HbA1c) showed that just sdLDL-C and HbA1c remained significantly associated with the risk of CVs. These results suggest that elevated Glucose and dyslipidemia might contribute to CVs.

**Table 4:** Predictors for cardiovascular events according to Cox^,^s proportional hazard analysis

Variable	Univariate model	Multivariate model	
	HR 95% CI	Model 1	Model 2
		HR 95% CI	HR 95% CI
Age	1.065 (1.136–1.624)**	1.034 (1.015–1.583)*	1.089 (1.025–1.590)**
Gender (men)	0.801 (0.456–1.564)	0.771 (0.469–1.693)	0.774 (0.501–1.763)
LDL-C (per 10 mg/dL)	1.103 (1.093–1.347)**	1.135 (0.857–1.432)	–
sdLDL-C (per 10 mg/dL)	1.285 (1.145–19.033)**	1.276 (1.201–16.664)**	1.281 (1.225–16.032)**
Non-HDL-C (per 10 mg/dL)	1.089 (1.038–1.945)**	1.131 (0.955–1.836)	–
RLP-C	1.165 (0.873–2.055)	–	–
Glucose	1.107 (1.066–1.208)*	0.996 (0.905–1.202)	–
HbA1c	1.305 (1.13–2.312)***	1.321 (1.142–2.406)**	1.225 (1.152–2.412)**
ApoA-I (per 10 mg/dL)	0.889 (0.762–0.941)**	0.892 (0.798–1.042)	–
ApoB (per 10 mg/dL)	1.125 (1.095–2.055)**	0.796 (0.718–1.944)	–

LDL-C: low-density lipoprotein cholesterol; sdLDL-C: small dense low-density lipoprotein cholesterol; HDL-C: high-density lipoprotein cholesterol; RLP-C: remnant lipoprotein cholesterol; HbA1c: hemoglobin A1c; ApoA-I: apolipoprotein A-I; ApoB: apolipoprotein B

The step-wise Cox regression and multivariate Cox regression analysis were used to regulate these independent predictors

^*^P < 0.05, ^**^P < 0.01, ^***^P < 0.001

Spearman's correlation analysis suggested that compared with LDL-C, the serum level of sdLDL-C exhibited more significant correlations with various parameters, suggesting that sdLDL-C might the major factor among LDL-C contribute to CVs.

**Fig. 2:**
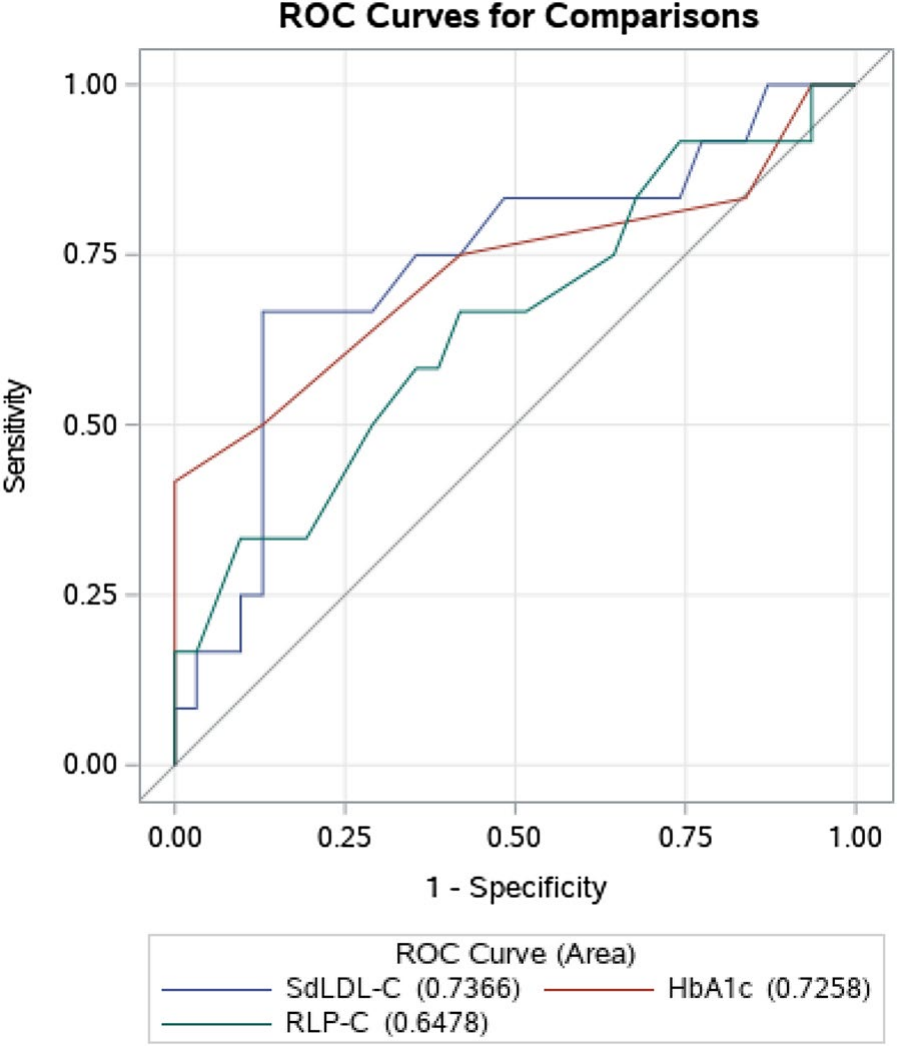
Receiver operating characteristic curves showed discriminatory power of sdLDL-C, HbA1C and RLP-C on CVs

We performed the ROC analysis in order to test the discriminatory power of sdLDL-C for the CVs (Fig. 2). The result indicate that the AUC of the sdLDL-C has a strong discriminating power against CVs, and its optimal cut-off value is 36.2mg/dL (AUC = 0.736, P = 0.003) than HbA1C and RLP-C.

## Discussion

Diabetes mellitus patients are often accompanied by dyslipidemia, which is the major controllable risk factor associated with cardiovascular disease (CVD) events [14]. Dyslipidemia has been confirmed as one of the principal processes underlying CVD, while sdLDL-C is considered as an emerging risk factor for CVD. Indeed, about 70% of elderly patients with type 2 DM die from CVD [15, 16].

SdLDL particles were defined as LDL with an average diameter of < 25.5 nm and had some features related to atherogenic potential: (1) increased penetration into the arterial wall by the small sizes, (2) a longer circulation time with low affinity for the LDL receptors, (3) high susceptibility to oxidation by containing little antioxidative vitamins [17]. Previous studies concerning the elevated levels of sdLDL-C has been associated with CVs [18, 19]. However, to the best of our knowledge, few studies have investigated on the influence of sdLDL-C levels on the onset of CVs in Chinese elderly patients with type2 DM.

In the present study, Spearman's rank correlation analysis suggest that sdLDL-C might the major factor among LDL-C contribute to CVs. Furthermore, ROC curve assay indicated that the AUC of the sdLDL-C has a strong discriminating power against CVs, and its optimal cut-off value is 36.2mg/dL (AUC=0.736, P = 0.003) than HbA1C and RLP-C (Fig. 2). Kaplan-Meier event-free survival curve displayed a obvious increase of CVs risk for the median levels of sdLDL‐C (Fig.1a). This phenomenon had analogous results in patients who received statins at baseline (Fig. 1b). Cox regression analysis showed that increase in sdLDL-C and HbA1c revealed a higher risk for CVs. From Table 5, we do see the correlation between sdLDL-C and RLP-C, but in the multivariable model, the RLP-C variable has been excluded from the regression automatically because less correlation with the outcome compare to sdLDL-C (Table 4).

**Table 5:** Spearman’s correlation of LDL-C and sdLDL-C to inflammatory biomarkers and age

Variables	LDL-C		sdLDL-C	
	r	P	r	P
age	− 0.194	0.009	− 0.164	0.025
Triglycerides	0.171	0.018	0.323	0.001
HDL-C	− 0.029	0.702	− 0.363	0.001
Non-HDL-C	0.563	0.001	0.588	0.001
RLP-C	0.406	0.011	0.521	0.001
HbA1c	0.024	0.802	0.061	0.431
ApoA-I	− 0.035	0.704	− 0.301	0.001
ApoB	0.622	0.001	0.623	0.001
Lp (a)	0.252	0.008	0.027	0.831

LDL-C: low-density lipoprotein cholesterol; sdLDL-C: small dense low-density lipoprotein cholesterol; HDL-C: high-density lipoprotein cholesterol; RLP-C: remnant lipoprotein cholesterol; HbA1c: hemoglobin A1c; ApoA-I: apolipoprotein A-I; ApoB: apolipoprotein B; Lp (a): lipoprotein (a)

For the variable not follow normal distribution, Spearman’s rank correlation usually was used

To determine whether sdLDL-C was an independent risk factor, we use the step-wise COX regression, even if the correlated variables are included, some of them will be excluded from the model automatically if they are not relevant compared to the investigational factor. The models were built after adjustment age, gender and CVs risk factors. Model 1 including Glucose, HbA1c, LDL-C, Non-HDL-C, sdLDL-C, ApoA-I and ApoB and Model 2 only including sdLDL-C and HbA1c showed that just sdLDL-C and HbA1c remained significantly associated with the risk of CVs These results suggest that elevated Glucose and dyslipidemia might contribute to CVs.

Indeed, sdLDL-C levels has the ability to predict CVD better than total LDL-C [18]. In addition, the Québec Cardiovascular Study has shown that sdLDL-C is interrelated with an raised risk of CAD in men [6, 7]. On the other hand, remnant lipoproteins are rich in TG and the main components include VLDL in the fasting state [20]. Obviously, the current study not only confirmed the sdLDL-C concentrations was an independent risk predictor for CVs, but also provided novel information concerning the role of RLP‐C in predicting CVs in diabetic patients [21–24].

In this study, both sdLDL-C and HbA1c are independent predictors for future cardiovascular events, and Table 5 shows that sdLDL-C and HbA1c are not significantly correlated. These inconsistent results may be partly due to HbA1c levels are stable over a period of time, while sdLDL-C is dynamically changing. Then, studies on the relationship between HbA1c and sdLDL-C at different time points over a period of time may present different results. Of course, sdLDL-C levels of type 2 DM patients are affected by many lipid-lowering agents used [25]. In order to manage type 2 DM patients better, it is necessary to analyze sdLDL-C in time to guide the health management of type 2 DM patients. This finding suggested that sdLDL-C should be focused on in the process of type 2 DM patients health management.

There are some limitations in this cohort study. First of all, the sample size is small and all the patients are Chinese, which may lead to a bias to fully observe the results and/or severity of CAD. Besides, of all the lipid biomarkers, only sdLDL-C was found independently associated with CVs among elderly patients with diabetes mellitus; whether it is because of the effect of statin therapy needs further investigation. Finally, the comparison of the predictive ability of sdLDL-C for CVs to the patient subgroup needs further research. Another limitation of the present study is that during the follow-up period the examinations of many patients were not collected and there were too many missing data; as a result of this, available data during the follow-up were not fully credible, which could easily lead to bias. Further limitation of the present study is that many patients had multiple antidiabetic drug combinations, and it was difficult to compare the detailed antidiabetic agents used.

## Conclusion

The current study demonstrated that sdLDL-C was an effective predictor in predicting the future CVs of elderly patients with type 2 DM and dyslipidemia.

## Data Availability

Unfortunately, the initial data cannot be shared as it contains confidential information.

## Abbreviations

DM: Diabetes mellitus
CVs: Cardiovascular events
CVD: Cardiovascular diseases
sdLDL-C: Small dense low-density lipoprotein cholesterol
RLP-C: Remnant lipoprotein cholesterol
LDL-C: Low-density lipoprotein cholesterol
HDL-C: High-density lipoprotein cholesterol
HbAlc: Hemoglobin A1c
Apo: Apolipoprotein
Lp (a): Lipoprotein (a)
ROC curves: Receiver operating characteristic curves
